# Erratum: Development and implementation of a Core Genome Multilocus Sequence Typing (cgMLST) scheme for Haemophilus influenzae

**DOI:** 10.1099/mgen.0.001304

**Published:** 2024-10-22

**Authors:** Made Ananda Krisna, Keith A. Jolley, William Monteith, Alexandra Boubour, Raph L. Hamers, Angela B. Brueggemann, Odile B. Harrison, Martin C. J. Maiden

**Affiliations:** 1 Nuffield Department of Medicine, Centre for Tropical Medicine and Global Health, University of Oxford, UK; 2 Department of Biology, University of Oxford, UK; 3 Oxford University Clinical Research Unit Indonesia, Faculty of Medicine Universitas Indonesia, Jakarta, Indonesia; 4 Department of Biology and Biochemistry, University of Bath, UK; 5 Nuffield Department of Population Health, University of Oxford, UK

In the published version of the article, there was a production error regarding the listed corresponding authors. Alexandra Barbour was incorrectly listed as one of the corresponding authors. The correct marking of the corresponding author can be found below:

Made Ananda Krisna^1,2,3^, Keith A. Jolley^2^, William Monteith^2,5^, Alexandra Boubour^4^, Raph L. Hamers^1,3^, Angela B. Brueggemann^4^, Odile B. Harrison^2,4,#^, Martin C. J. Maiden^2^

# Correspondence: Odile B. Harrison

odile.harrison@ndph.ox.ac.uk

Author affiliations

1. Nuffield Department of Medicine, Centre for Tropical Medicine and Global Health, University of Oxford, UK.
2. Department of Biology, University of Oxford, UK.
3. Oxford University Clinical Research Unit Indonesia, Faculty of Medicine Universitas Indonesia, Jakarta, Indonesia.
4. Nuffield Department of Population Health, University of Oxford, UK.
5. Department of Biology and Biochemistry, University of Bath, UK.

The Microbiology Society apologises for any inconvenience caused.

